# Fibromyalgia: are you a genetic/environmental disease?

**DOI:** 10.1097/PR9.0000000000001256

**Published:** 2025-04-18

**Authors:** Jacob N. Ablin

**Affiliations:** Department of Medicine H, Tel Aviv Sourasky Medical Center & Faculty of Medicine, Tel Aviv University, Tel Aviv, Israel

**Keywords:** Fibromyalgia, Genetic predisposition, Environmental factors, Epigenetics, Nociplastic pain

## Abstract

Fibromyalgia, characterized by chronic widespread pain and fatigue, involves complex interactions between genetic predispositions and environmental triggers. This review delves into the multifaceted nature of fibromyalgia, emphasizing recent advances in understanding its pathogenesis through genetic, epigenetic, and environmental lenses. We explore the roles of specific genetic polymorphisms, such as those in the catechol-O-methyltransferase and serotonin transporter genes, and their correlation with the syndrome's susceptibility. The review also examines the significant impact of environmental factors, including physical trauma and stress, which potentiate the syndrome's severity. In addition, emerging research on the microbiome and epigenetic modifications provides new insights into the disease mechanisms, potentially guiding future therapeutic strategies. This article aims to synthesize current research findings and propose directions for future research, underscoring the necessity of a multidisciplinary approach to decipher the complexities of fibromyalgia.

## Abstract 2

Fibromyalgia, influenced by genetic and environmental factors, requires a multidisciplinary approach. Advances in genetics, microbiome research, and AI promise improved diagnosis and treatment.

## 1. Introduction

The syndrome of fibromyalgia, characterized by chronic widespread pain and fatigue, is increasingly recognized as one of the major challenges in the world of pain. Although on the one hand straightforward in its cardinal clinical manifestations, and thus theoretically easy enough to recognize and diagnose, fibromyalgia continues to attract heated debate and controversy from many quarters, much to the detriment of the individuals suffering from this complex condition. Much of the frustration confronted by patients with fibromyalgia, as well as by many health care professionals attempting to care for these patients, focuses on the subjective nature of the condition and the lack of readily available objective biomarkers that could serve to validate the suffering reported by patients and to disperse mistrust and accusations of malingering on the part of the medical team. This unfortunate situation has not changed over the past decades despite increasing volumes of research, which has shed light on the neuroscience and pathophysiology of fibromyalgia.^19,50^ Moreover, the medicolegal aspects related to this chronic, often debilitating condition, which frequently affects young individuals at their most productive years of life, are similarly marked by ongoing controversy and often acrid dispute, relating inter alia to the etiological role of various environmental triggers, including physical trauma (especially motor vehicle accidents) and emotional stress.^15,53,75^ In view of these challenges, there is considerable practical demand for a better understanding of the pathogenesis of fibromyalgia, which would lead to more objective and precise diagnostic tools as well as biomarkers of disease severity.

A strong familial component of fibromyalgia has been recognized for many years,^5,6^ and this has naturally led to rather extensive investigations into the genetic underpinnings of the syndrome. These efforts have evolved over time, hand in hand with the evolution of modern genetics. Initial studies used hypothesis-based strategies to focus on specific target genes, considered to play a role in the transmission and processing of pain. Although these efforts have met with some success, the introduction of more advanced genetic tools such as genome-wide association study (GWAS) has naturally led the way into a novel era in the investigation of fibromyalgia genetics, zooming out from the individual single nucleotide polymorphism (SNP) and attempting to describe the broader intricate puzzle of genetic traits, which may be responsible for an individual's susceptibility for developing fibromyalgia (and chronic pain in general).^1,26,27,64,68^ More recently evolving tools such as machine learning (ML) are being harnessed in the attempt to analyze these vast data sets of genetic information.^51,78^ However, even with all the progress made in describing genetic information, it remains self-evident that complex conditions such as fibromyalgia cannot be explained by genetics alone. An intricate set of environmental triggers has been associated with the condition, including but not limited to physical trauma, infection, and various models of stress.^3,13,43,59^ One particularly recent and striking piece that has been added to the puzzle is related to the case of COVID-19, which has evolved into the long COVID syndrome, soon recognized as overlapping considerably with fibromyalgia.^18,66^ Similar to other aspects of the COVID-19 pandemic that have been studied on the genetic level, such as identifying those individuals prone to develop severe COVID-19 and respiratory failure, the interaction between genetic predisposition and the development of post–COVID-19 symptoms evolving into a fibromyalgia-like condition is a classic example of the interaction between genetics and environmental factors, along the road to pathogenesis. Another fascinating venue where these aspects may meet in relation to fibromyalgia is related to the field of microbiome research. As increasing research points toward the role played by the microbiome in the pathogenesis of fibromyalgia and even the possible therapeutic potential of microbiome-manipulation,^16,54^ once again the question is raised regarding to what extent an interaction between genetic predisposition and the microbiome might play a role. Moreover, significant progress in the field of epigenetics promises to bridge the gap between genetic predisposition, environmental triggers, and the ultimate development of fibromyalgia.^60^

Fibromyalgia is a complex condition, considered to be explained mainly in an alteration in the way the nervous system processes pain, and currently described in nociplastic pain.^30^ Thus, similar to other complex multifactorial conditions affecting the nervous system, a multifaceted approach must be adopted and sequentially refined to eventually reach a more comprehensive understanding and with the hope of achieving improved tools for diagnosis and treatment. In the following article, we will attempt to cover the current aspects of this conundrum and point toward future directions.

**Figure 1.**
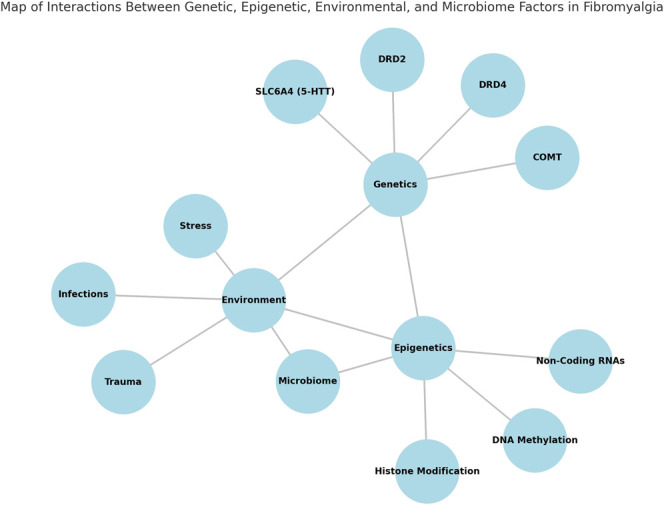
Map of interactions between genetic, epigenetic, environmental, and microbiome factors in fibromyalgia.

The interplay between genetics, environment, epigenetics, and microbiome is central to understanding fibromyalgia. Figure 1 illustrates these interconnected factors, providing a conceptual map of their contributions to the condition.

## 2. Methodology for data collection and review

To provide a comprehensive and evidence-based narrative review of fibromyalgia, we conducted a structured search of peer-reviewed scientific literature across multiple databases. The following methodology was used to ensure transparency and rigor:

### 2.1. Databases used

The primary databases consulted were:

(1) PubMed: For biomedical and life sciences research.
(2) Scopus: For multidisciplinary articles with a focus on genetics, epigenetics, and pain research.
(3) Web of Science: For highly cited articles and systematic reviews relevant to fibromyalgia.
(4) Embase: For European literature and pharmacological studies.
(5) Google Scholar: To capture gray literature and recent publications not yet indexed in other databases.

### 2.2. Criteria for study inclusion

To ensure relevance and quality, the following inclusion criteria were applied:

Publication date: Articles published from January 2000 to December 2023.
Language: Only English-language articles were included.
Study type: Focus on narrative reviews, systematic reviews, meta-analyses, clinical trials, and observational studies.
Topics: Articles discussing fibromyalgia pathophysiology, genetic and epigenetic mechanisms, environmental factors, microbiome, nociplastic pain, and the application of artificial intelligence (AI).
Population: Studies involving adult patients with fibromyalgia, with exclusions for pediatric studies unless highly relevant.

### 2.3. Search strategy

A combination of Medical Subject Headings terms and free-text keywords was used, including: “Fibromyalgia,” “Genetics of fibromyalgia,” “Epigenetics,” “Environmental triggers,” “Microbiome and fibromyalgia,” and “Artificial intelligence in chronic pain.”

### 2.4. Research period

The search focused on studies published within the past 20 years to capture contemporary advancements in fibromyalgia research. Foundational studies older than this period were included only if they were highly cited and seminal to the field.

### 2.5. Data extraction and analysis

The extracted studies were analyzed for quality, relevance, and methodological rigor. Attention was given to the sample size, study design, and limitations of each study to ensure an accurate synthesis of findings.

## 3. Genetics of fibromyalgia

As pointed out in the introduction, a strong familial association has been clearly documented in the fibromyalgia syndrome with the odds ratio of an individual developing fibromyalgia once a first degree proband has been diagnosed being as high as 1:9.^6^ Obviously, familial association is not directly equivalent with genetic association because shared environmental influences (including intrauterine effects) may play a role.^22,72^ Nonetheless, such strong familial association, which has also been supported by twin studies,^52,58^ leads to the assumption that at least part of the association must be attributed to genetic factors. Incidentally, this recognition has subsequently found its way into the clinical assessments of nociplastic pain, where a familial history of chronic pain syndromes has been recognized as supporting the diagnosis. Starting out from this familial recognition, early heritable studies in the field of fibromyalgia have attempted to investigate which genetic markers contribute to the variation in susceptibility to fibromyalgia within the population. Genetic markers are specific sequences in the genome that can be associated with increased risk of disease. Thus, genetic polymorphisms have been studied, which are considered to play a possible role in pain transmission and sensitivity, stress response, and inflammation.

Several studies have focused on the catechol-O-methyltransferase (COMT) gene, because of the assumption that this enzyme which is responsible for the metabolism of catecholamines may carry genetic variations that might affect the processing of pain.^47^

### 3.1. Role of catechol-O-methyltransferase in pain perception

The COMT enzyme degrades catecholamines both in the brain as well as in the peripheral nervous system.^56^ Thus, its activity may influence pain perception, stress reactivity, and mood regulation.^71^ Variations in COMT activity can affect the levels of these neurotransmitters, potentially altering an individual's pain sensitivity and stress response. High COMT activity results in lower levels of dopamine, possibly leading to increased pain perception and a lower threshold for pain.^76^ Conversely, lower COMT activity may lead to higher dopamine levels, which may be protective against pain but could also affect mood and emotional responses.^69^

### 3.2. Catechol-O-methyltransferase gene polymorphisms and fibromyalgia

The most studied polymorphism in the context of fibromyalgia has been Val158Met (rs4680), where a substitution of methionine (Met) for valine (Val) at codon 158 results in reduced COMT enzyme activity.^63^ Individuals with the Met/Met genotype have been shown to have lower COMT activity, higher dopamine levels, and potentially increased pain sensitivity compared with those with the Val/Val genotype.^20^ Research has indicated that the Met allele may be associated with an increased risk of fibromyalgia, as well as being associated with higher pain sensitivity among patients.^37^ Studies have found that patients with fibromyalgia with the Met/Met genotype report greater pain intensity, more tender points, and heightened sensitivity to painful stimuli compared with those carrying the Val allele. Notably, the impact of COMT polymorphisms on pain perception may be influenced by environmental factors, such as stress and trauma. Individuals with the Met/Met genotype might be more susceptible to developing fibromyalgia after physical or emotional stress, suggesting a gene–environment interaction in the pathogenesis of the condition.^67^ Understanding the role of COMT polymorphisms in fibromyalgia could have implications for diagnosis and treatment. Genetic testing for COMT variants may help identify individuals at increased risk for fibromyalgia or those who may benefit from specific therapeutic approaches targeting the dopaminergic system.

### 3.3. Serotonin transporter gene polymorphisms

Serotonin has long been recognized as a key neurotransmitter involved in mood regulation, pain perception, and the stress response. The serotonin transporter gene (SLC6A4) encodes the serotonin transporter (5-HTT), crucial for serotonin reuptake in the brain.^38^ A well-studied polymorphism in the 5-HTT gene is the serotonin transporter–linked polymorphic region (5-HTTLPR), which includes long (L) and short (S) allele variations.^41^ The S allele is associated with reduced efficiency of serotonin uptake, potentially leading to altered pain perception and increased risk of mood disorders.^34^ Studies suggest that individuals with the S allele may exhibit increased sensitivity to pain, a hallmark of fibromyalgia.^49^ This increased pain sensitivity is believed to result from altered serotonergic signaling within the central nervous system. Given the role of serotonin in mood regulation, 5-HTTLPR polymorphisms have also been explored in the context of comorbid mood disorders in patients with fibromyalgia. The S allele has been linked to a higher prevalence of anxiety and depression among these individuals.^8^

### 3.4. Dopamine receptor gene polymorphisms

Dopamine plays a crucial role in reward, motivation, pain processing, and the regulation of movement. Dopamine receptors, encoded by several genes (eg, DRD1, DRD2, DRD3, DRD4, and DRD5), mediate the effects of dopamine in the nervous system.^31^ Variations in dopamine receptor genes, particularly DRD2 and DRD4, have been investigated for their association with fibromyalgia.^14^ These polymorphisms may affect receptor function and dopamine signaling pathways, influencing pain processing and stress reactivity. Polymorphisms in dopamine receptor genes might contribute to the altered pain perception and dysfunctional stress response observed in patients with fibromyalgia. For example, certain DRD2 polymorphisms have been associated with increased pain sensitivity and altered responses to stress.^25^ Understanding these genetic variations holds the potential to inform therapeutic targets. For instance, treatments that modulate dopaminergic pathways might be more effective in individuals with specific DRD polymorphisms, pointing toward personalized medicine approaches in fibromyalgia management.

Table 1 lists some of the most well-studied polymorphisms associated with the fibromyalgia syndrome.

**Table 1:** Comparative table of genetic polymorphisms in fibromyalgia.

Gene	Polymorphism	Effect	Clinical relevance
COMT	Val158Met (rs4680)	Influences pain sensitivity through dopamine regulation	Increased pain sensitivity in Met/Met genotype
SLC6A4 (5-HTT)	5-HTTLPR	Affects serotonin uptake, linked to pain and mood disorders	S allele linked to higher pain sensitivity
DRD2	Taq1A	Modulates dopamine receptor function, associated with stress response	Associated with chronic pain conditions
DRD4	48-bp VNTR	Regulates novelty-seeking traits and stress reactivity	Potential link to pain perception and fibromyalgia risk

5-HTT, serotonin transporter; 5-HTTLPR, serotonin transporter–linked polymorphic region; COMT, catechol-O-methyltransferase; Met, methionine.

### 3.5. Genome-wide association studies in fibromyalgia research

Genome-wide association study (GWAS) is a method that allows researchers to examine the genome-wide set of genetic variants in different individuals to see whether any variant is associated with a trait, such as susceptibility to fibromyalgia and chronic pain.^26,62,64^ This is particularly useful for diseases with complex etiologies that likely result from the interplay of multiple genetic and environmental factors. This is achieved through the use of SNP chips that can assess hundreds of thousands to millions of SNPs across the genome in large populations. As implemented in fibromyalgia, GWAS has identified genes involved in the dopaminergic and serotonergic pathways, as well as in inflammatory and stress response–related systems.

One of the main challenges with GWAS findings in fibromyalgia has been the need for replication in independent cohorts. Given the heterogeneity of the condition and the small effect sizes of individual genetic variants, large-scale studies are required to validate these associations. Future research might benefit from combining GWAS findings with other “omic” technologies, such as transcriptomics, proteomics, and metabolomics. Such integrative analyses may provide a more comprehensive view of the biological pathways involved and the ways in which genetic variations influence these pathways. In addition, there is a need for functional studies to understand how identified variants affect biological functions and contribute to fibromyalgia pathophysiology.

The insights into fibromyalgia's genetic basis, particularly from studies on COMT, serotonin transporter, and dopamine receptor polymorphisms, provide a strong foundation for understanding individual susceptibility to fibromyalgia. However, the complexity of the condition necessitates integrating genetic findings with environmental and epigenetic insights to paint a complete picture of its pathogenesis. This sets the stage for exploring how external factors may interact with these genetic predispositions, as discussed in the following sections.

## 4. Environmental factors in fibromyalgia

In the complex landscape of fibromyalgia, environmental influences play a pivotal role in the onset, progression, as well as the severity of the condition. This section explores the multifaceted impact of external factors, from stress and lifestyle to broader socioeconomic determinants, on fibromyalgia.

### 4.1. Stress factors

Patients with fibromyalgia often report a heightened sensitivity to stress, which can exacerbate symptoms. Physical stressors, such as injury or illness, alongside emotional stressors such as anxiety and depression, significantly contribute to the disease's severity. Chronic stress due to lifestyle or environmental changes, notably during the COVID-19 pandemic, has been shown to disrupt the daily lives of patients, increasing their symptomatology. The pandemic served as a global stressor, affecting mental health and access to care for patients with fibromyalgia, thereby intensifying their levels of pain and fatigue.^4^ It is difficult to precisely decipher the role of viral infection per se, as opposed to the various accompanying effects of the pandemic, but it is noteworthy that fibromyalgia has previously been associated with other infectious triggers, most notably Epstein–Barr virus.^2^

### 4.2. Lifestyle factors

#### 4.2.1. Diet and nutrition

Nutrition may play an important role in managing fibromyalgia symptoms. Diets rich in fruits, vegetables, and lean proteins, while low in processed foods, may mitigate inflammation and reduce pain.^4^ Conversely, certain foods may trigger flare-ups, underscoring the need for personalized dietary plans.^42,65^

#### 4.2.2. Physical activity

Regular, moderate exercise is beneficial in fibromyalgia management, aiding in pain reduction and improving sleep quality. However, owing to the tendency toward exercise intolerance, patients face a challenge in balancing activity levels to avoid exacerbating their symptoms.^28,79^

#### 4.2.3. Socioeconomic status

Access to health care, employment stability, and educational background significantly influence fibromyalgia outcomes.^7,73^ Disparities in health care access and the socioeconomic burden of the disease can hinder effective management and treatment adherence.

### 4.3. Nociplastic pain: bridging the genetic–environmental divide

Nociplastic pain, characterized by altered nociception in the absence of clear tissue damage, is a hallmark of fibromyalgia.^30^ This section delves into how genetic predispositions and environmental factors converge to manifest nociplastic pain. Environmental triggers, such as infections or ongoing stress, may activate latent genetic susceptibilities, leading to the development or exacerbation of fibromyalgia symptoms. The interplay between the microbiome and epigenetics presents another intriguing area of research. Preliminary studies suggest that alterations in the gut microbiome may influence fibromyalgia symptoms,^54,55^ potentially mediated through epigenetic mechanisms. Intriguing research recently published by Minerbi et al.^16^ has demonstrated that fibromyalgia-like symptoms can be transferred from patients to mice, by way of fecal transplantation and reversed after transplantation from norma individuals. The researchers also conducted a pilot trial in humans, demonstrating the positive clinical effects of fecal transplantation in patients with fibromyalgia. These findings highlight the intricate connections between our environment, our genes, and their expression, potentially offering novel avenues for understanding and treating fibromyalgia. The significant role of environmental factors, from stress and trauma to lifestyle influences, underscores the importance of external triggers in the onset and progression of fibromyalgia. Although these factors independently affect symptom severity, their interplay with genetic predispositions highlights the need for a multifactorial approach to treatment and prevention. This interplay is further elucidated through the lens of epigenetics, as explored in the next section.

## 5. Epigenetics

Epigenetics, the study of heritable changes in gene expression, that do not involve alterations to the underlying DNA sequence offers profound insights into the complex interplay between genes and the environment.^80^ In the context of fibromyalgia, epigenetic mechanisms such as DNA methylation, histone modification, and noncoding RNA activity are increasingly recognized for their role in modulating pain perception, stress responses, and inflammation, possibly contributing to the disease's pathophysiology and symptom variability among patients.

### 5.1. Epigenetic mechanisms

#### 5.1.1. DNA methylation

This process involves the addition of a methyl group to the DNA molecule, typically at cytosine–phosphate–guanine sites, leading to gene silencing.^10^ In fibromyalgia, aberrant DNA methylation patterns may downregulate or upregulate genes associated with pain sensitivity, immune function, and stress responses, potentially influencing disease susceptibility and the severity of symptoms.^32,33^

#### 5.1.2. Histone modification

Histones are proteins around which DNA winds, and their chemical modifications can influence gene expression.^44^ Modifications such as acetylation and methylation can facilitate or inhibit the transcription of genes involved in pain pathways, immune regulation, and stress resilience.^40,70^ Altered histone modification patterns in patients with fibromyalgia could thus contribute to the dysregulation of these critical biological processes.^24^

#### 5.1.3. Noncoding RNA

Noncoding RNAs, including microRNAs and long noncoding RNAs, regulate gene expression at the post-transcriptional level, affecting various cellular processes.^12,21^ In fibromyalgia, specific noncoding RNAs might target messenger RNAs involved in pain signaling, immune responses, and neuroendocrine functions, thereby modulating the disease phenotype.^11^

### 5.2. Gene–environment–epigenetic interactions

The interplay between genetic predispositions, environmental exposures, and epigenetic modifications underscores the multifactorial nature of fibromyalgia. Stress, diet, physical activity, and exposure to toxins or infections may lead to epigenetic changes that activate or silence genes associated with fibromyalgia risk and symptomatology. For instance, chronic stress may alter DNA methylation patterns related to the hypothalamic–pituitary–adrenal axis, affecting stress hormone levels and pain sensitivity.^46,61^ Furthermore, the reversibility of epigenetic modifications presents a potential avenue for therapeutic intervention. Understanding how specific environmental factors induce epigenetic changes offers the possibility of modulating these factors or directly targeting epigenetic mechanisms to alleviate fibromyalgia symptoms.

Although the exploration of epigenetics in fibromyalgia is still in its infancy, this field holds the promise of unlocking new diagnostic tools, therapeutic targets, and personalized treatment strategies. Future research should focus on identifying epigenetic biomarkers that can predict disease risk, progression, and response to treatment, facilitating early intervention and tailored therapy plans.

Epigenetic therapies, including drugs that modulate DNA methylation and histone modification patterns, are already being explored for other conditions, especially in oncology^9^ and may be applicable to fibromyalgia. In addition, lifestyle interventions, such as stress reduction techniques, dietary adjustments, and physical activity, may influence epigenetic profiles, offering a nonpharmacological approach to managing disease. Thus, the role of epigenetics in fibromyalgia underscores the dynamic relationship between genes and environment, highlighting the potential for innovative approaches to treatment that leverage the growing understanding of these complex mechanisms. As research advances, the field of epigenetics is poised to play a crucial role in unraveling the fibromyalgia conundrum, paving the way for more effective management and improved outcomes.

Epigenetic research bridges the gap between genetic predispositions and environmental triggers, revealing dynamic modifications that influence fibromyalgia risk and symptomatology. The reversibility of epigenetic changes offers hope for novel therapeutic interventions. These findings invite deeper exploration of related mechanisms, such as the microbiome, which might act as both an environmental and epigenetic modulator, as subsequently discussed.

The Human Phenotype Project, as developed by the Weizmann Institute, represents an ambitious endeavor to catalog and understand the vast diversity of human phenotypes—the observable traits and characteristics influenced by both genetic and environmental factors.^48^ This project's expansive approach to collecting and analyzing phenotypic data holds significant promise for advancing our understanding of complex conditions such as fibromyalgia, which is characterized by a wide range of symptoms and comorbidities that vary greatly among patients. The project aims to create a comprehensive database that links detailed phenotypic descriptions with underlying genetic information. By using a standardized vocabulary and classification system, the project facilitates the precise identification and analysis of phenotypes across individuals and populations. This meticulous approach enables researchers to dissect the genetic and environmental underpinnings of diseases, including multifaceted conditions such as fibromyalgia.

Fibromyalgia, with its complex symptomatology and unclear etiology, stands to benefit immensely from the insights generated by the Human Phenotype Project and similar endeavors. The project's detailed phenotypic data could help identify specific patterns and clusters of symptoms, revealing subtypes of fibromyalgia that may have distinct genetic and environmental causes. This stratification is crucial for developing targeted treatment strategies and understanding the disease's diverse manifestations.

Moreover, the project's integration of environmental data with phenotypic and genetic information allows for a nuanced analysis of how external factors contribute to fibromyalgia. For example, it can shed light on the impact of stress, diet, and physical activity on symptom severity and disease progression. This holistic view of fibromyalgia can guide personalized interventions and inform public health strategies aimed at mitigating environmental risk factors.

### 5.3. Insights into gene–environment interactions

A unique strength of the Human Phenotype Project is its potential to elucidate gene–environment interactions that play a role in fibromyalgia. By correlating specific environmental exposures with phenotypic outcomes in genetically characterized individuals, the project can identify environmental factors that exacerbate or mitigate the risk of developing conditions such as fibromyalgia in the presence of certain genetic predispositions. This understanding of gene–environment interplay is instrumental in recognizing the triggers of fibromyalgia and predicting individual susceptibility to the syndrome. It paves the way for preventive measures and early interventions, tailored to the genetic and environmental context of each patient, moving closer to the holy grail of precision medicine in fibromyalgia care.

The ambition of the Human Phenotype Project and similar efforts bring with it a set of challenges, including the need for vast data sets, sophisticated data analysis tools, and robust privacy protections. Moreover, the dynamic nature of phenotypes, which can change over time and in response to various factors, adds complexity to their analysis.

Despite these challenges, the project's contributions to the research of complex and multifactorial entities such as fibromyalgia are poised to be transformative. By providing a detailed map of the phenotypic landscape of fibromyalgia, the Human Phenotype Project may help to unravel the complexities of the syndrome. It offers hope for more effective treatments, improved patient outcomes, and a deeper understanding of the intricate dance between our genes and our environment. As the project progresses, it will undoubtedly continue to shed light on the mysteries of fibromyalgia and other multifactorial diseases, marking a significant advancement in the field of human health and disease.

### 5.4. The Research Domain Criteria framework: a novel paradigm for understanding fibromyalgia

The Research Domain Criteria (RDoC) framework, developed by the National Institute of Mental Health, represents a groundbreaking shift in the approach to understanding and researching mental illnesses.^23^ Moving beyond the traditional symptom-based diagnostic criteria exemplified by the Diagnostic and Statistical Manual of Mental Disorders (DSM), RDoC proposes a biologically based framework that focuses on understanding the underlying mechanisms of mental disorders.^29^ This approach is particularly relevant to conditions such as fibromyalgia, which, despite being primarily characterized as a chronic pain condition, encompasses significant psychological and neurobiological components that overlap with mental health disorders.

### 5.5. Research Domain Criteria framework vs Diagnostic and Statistical Manual of Mental Disorders: a paradigm shift

The DSM categorizes mental health disorders based on clusters of symptoms and behaviors, facilitating diagnosis and treatment. However, this symptom-based approach often overlooks the underlying biological, genetic, and environmental factors contributing to the disorders. By contrast, the RDoC framework seeks to integrate multiple levels of information (from genes to neural circuits to behaviors) to understand mental health conditions more comprehensively.^45^ It emphasizes the importance of dimensional aspects of functioning across different domains, such as cognitive systems, negative and positive valence systems, and systems for social processes, rather than discrete categories of illness.

### 5.6. Applicability to fibromyalgia

Fibromyalgia, traditionally diagnosed through symptom-based criteria established by the American College of Rheumatology,^74^ which include widespread pain and tenderness in specific points across the body, suffers from similar limitations as the DSM in mental health. These criteria are inherently subjective and have undergone revisions, reflecting the challenges in capturing the complex nature of fibromyalgia through symptomatology alone.

The RDoC framework offers an alternative approach by suggesting that fibromyalgia, like many mental health disorders, could be better understood by examining the underlying biological, psychological, and social dimensions that contribute to the condition. For instance, fibromyalgia's association with dysregulated pain processing, mood disorders, and cognitive impairments could be explored through the RDoC domains, such as:

(1) Negative valence systems: Investigating the mechanisms of heightened pain perception and the co-occurrence of anxiety and depression in patients with fibromyalgia.
(2) Cognitive systems: Understanding deficits in attention, memory, and executive function among fibromyalgia sufferers.
(3) Systems for social processes: Examining the impact of fibromyalgia on social interactions and perceptions of social support, which are critical for patient outcomes.

By adopting the RDoC framework, research into fibromyalgia can move beyond the limitations of symptom-based diagnostic criteria and explore the multifaceted biopsychosocial model of the disease. This approach could enable a more nuanced understanding of fibromyalgia's etiology, potentially leading to targeted interventions that address the underlying mechanisms, rather than just alleviating symptoms.

### 5.7. Implications for diagnosis and treatment

Transitioning to a RDoC-based understanding of fibromyalgia could revolutionize diagnostic processes, making them considerably more objective and biologically grounded. It could also facilitate the development of personalized treatment strategies that consider the unique biological and psychological profile of each patient. For instance, interventions could be tailored to target specific dysfunctions in neural circuits involved in pain perception, combined with the patients genetic profile, or to address particular cognitive or emotional challenges faced by a specific patient.

Integrating the RDoC framework into fibromyalgia research and treatment requires a concerted effort to collect and analyze data across the spectrum of genetic, biological, psychological, and social domains. It also necessitates a paradigm shift in the way in which patients, clinicians, and researchers conceptualize the condition. As this framework gains traction, it promises to uncover novel insights into fibromyalgia and pave the way for more effective, evidence-based approaches to managing this complex condition. By focusing on the underlying dimensions of functioning and pathology, the RDoC framework offers a promising path toward unraveling the complexities of fibromyalgia, moving beyond symptom management to address the root causes of the syndrome.

## 6. Advancements in artificial intelligence and machine learning: revolutionizing fibromyalgia research, diagnosis, and treatment

The integration of AI and ML technologies into the field of fibromyalgia represents a pivotal shift in the way in which this condition is studied, diagnosed, and treated.^51,77^ These technologies offer the potential to decode the complexity of fibromyalgia by analyzing vast data sets, identifying patterns, and predicting outcomes in ways that were previously unattainable. Their application spans from uncovering novel insights into the disease's etiology to personalizing patient care, marking a new era in the management of fibromyalgia.

### 6.1. Enhancing research with artificial intelligence and machine learning

Artificial intelligence and ML can process and analyze large-scale biomedical data, including genetic, epigenetic, phenotypic, and environmental information, to identify risk factors and potential therapeutic targets for fibromyalgia. By applying advanced algorithms to patient data, researchers can discover correlations and causations that elude traditional statistical methods. For instance, ML models can analyze data from wearable devices to find patterns in physical activity, sleep quality, and physiological responses that correlate with fibromyalgia flare-ups, offering insights into disease triggers and progression.^17,57^ The diagnostic process for fibromyalgia, traditionally reliant on subjective assessments and symptom-based criteria, can be significantly enhanced with AI. Machine learning algorithms, trained on diverse patient data, including clinical, laboratory, and imaging findings, can help identify biomarkers and symptom patterns specific to fibromyalgia. This approach could lead to the development of diagnostic models that are both objective and sensitive, reducing the time to diagnosis and minimizing the misdiagnosis rate.

Artificial intelligence–driven tools can also integrate data from electronic health records, patient-reported outcomes, and real-time monitoring to provide a holistic view of the patient's condition. This comprehensive approach enables the identification of fibromyalgia subtypes, facilitating personalized treatment plans that address the unique manifestations of the disease in each patient.^35,39^

### 6.2. Personalizing treatment through artificial intelligence and machine learning

The treatment of fibromyalgia can greatly benefit from AI and ML by moving toward precision medicine, where interventions are tailored to the individual's genetic makeup, lifestyle, and disease characteristics. Artificial intelligence models can predict individual responses to different therapies, including medications, physical therapy, and cognitive–behavioral therapy, based on historical data from similar patients and even on functional neuroimaging.^36^ This predictive capability can guide clinicians in selecting the most effective treatments, optimizing therapeutic outcomes, and minimizing adverse effects. Furthermore, ML algorithms can monitor patient responses to treatment in real-time, using data from wearable devices and mobile health applications. This continuous monitoring allows for dynamic adjustments to treatment plans, ensuring that interventions remain aligned with the patient's evolving needs and preferences.

As AI and ML technologies continue to advance, their potential to transform the field of fibromyalgia research and care grows. Future developments could include more sophisticated models for predicting disease onset, progression, and response to treatment, as well as AI-driven platforms for patient education and self-management.

However, the successful implementation of AI and ML in fibromyalgia faces several challenges, including the need for high-quality, standardized data, concerns regarding patient privacy and data security, and the requirement for interdisciplinary collaboration among computer scientists, clinicians, and researchers. Moreover, there is a need for regulatory frameworks that ensure the ethical use of AI in health care and the validation of AI-driven tools for clinical use. The advancements in AI and ML offer unprecedented opportunities to enhance our understanding, diagnosis, and treatment of fibromyalgia. By harnessing the power of these technologies, the medical community can move closer to delivering personalized, effective care to those affected by this complex condition.

### 6.3. Limitations of current research and future directions

Although this review highlights significant advancements in our understanding of fibromyalgia, several limitations in the current body of literature merit discussion. Genetic studies often lack replication across diverse populations, limiting their generalizability. Similarly, environmental studies rely heavily on retrospective designs, introducing potential biases. Epigenetic research, although promising, remains largely exploratory, with insufficient longitudinal data to establish causality. Microbiome studies face methodological inconsistencies, and the findings are often confounded by external factors such as diet. Finally, the use of AI in fibromyalgia research is still in its infancy, constrained by the quality and diversity of available data.

To address these gaps, future research should adopt a more integrative approach, combining genetic, epigenetic, environmental, and microbiome data. Larger, multicenter studies with standardized methodologies are essential to validate findings. Moreover, the development of longitudinal studies and high-quality data sets will help clarify causal relationships and advance precision medicine in fibromyalgia care.

The integration of AI and ML into fibromyalgia research and care holds immense promise for refining diagnosis, predicting disease trajectories, and personalizing treatment. As these technologies mature, their ability to synthesize genetic, epigenetic, environmental, and microbiome data will transform our understanding of fibromyalgia and its management. The practical implications of these findings for clinicians and patients are explored in the subsequent section.

## 7. Practical applications for clinicians and patients

The insights gained from genetic, environmental, and epigenetic research into fibromyalgia have the potential to transform clinical practice and improve patient outcomes. Below are key applications for clinicians and patients:

### 7.1. Personalized medicine in fibromyalgia care

Advances in understanding genetic polymorphisms, such as those in the COMT and serotonin transporter genes, can help stratify patients based on their genetic risk profiles. Clinicians may use genetic screening to:

(1) Identify patients with increased susceptibility to fibromyalgia.
(2) Tailor treatments targeting specific neurotransmitter systems, such as serotonin or dopamine modulation, to align with the patient's genetic makeup.

### 7.2. Guidance for nonpharmacological interventions

(1) Recognizing the significant role of environmental triggers, including stress and lifestyle factors, can guide the implementation of personalized, nonpharmacological interventions.
(2) Clinicians can recommend stress management strategies, cognitive–behavioral therapy, or graded exercise programs tailored to the individual's stress response profile.

### 7.3. Use of epigenetic insights

(1) The reversible nature of epigenetic modifications, such as DNA methylation and histone acetylation, opens avenues for targeted interventions. Lifestyle adjustments, such as improved nutrition, stress reduction, and regular exercise, may influence epigenetic profiles and mitigate symptoms.
(2) Future developments in epigenetic therapies could lead to novel pharmacological approaches.

### 7.4. Microbiome-based therapies

(1) Emerging findings on the microbiome's role in fibromyalgia pathogenesis suggest that clinicians could explore probiotic or fecal microbiota transplantation therapies for symptom management.
(2) Educating patients on dietary modifications to support a healthy microbiome may be a cost-effective, accessible intervention.

### 7.5. Application of artificial intelligence

(1) Artificial intelligence–driven tools, such as ML algorithms, can enhance the diagnostic process by analyzing diverse patient data and identifying subtle symptom patterns. This can help clinicians diagnose fibromyalgia earlier and more accurately.
(2) Artificial intelligence models may also predict treatment responses, allowing clinicians to personalize therapy based on historical data and patient characteristics, thereby optimizing therapeutic outcomes.

### 7.6. Empowering patients through education

(1) Knowledge of the interplay between genetics and environment can empower patients to adopt healthier behaviors and actively participate in their care plans.
(2) Clinicians can provide tailored education on managing environmental triggers, such as stress and poor sleep hygiene, to improve symptom control.

### 7.7. Multidisciplinary care models

(1) The multifactorial nature of fibromyalgia underscores the need for a collaborative approach involving rheumatologists, neurologists, psychologists, and physiotherapists.
(2) This integration ensures holistic care addressing not only pain but also comorbid conditions such as anxiety and depression, thereby enhancing patients' quality of life.

## 8. Conclusion: unraveling the complex tapestry of fibromyalgia

This review has traversed the multifaceted landscape of fibromyalgia, shedding light on the intricate interplay between genetic predispositions and environmental influences that underpin this enigmatic condition. The exploration of genetic markers, heritability studies, and gene–environment interactions has illuminated the foundational role of genetics in predisposing individuals to fibromyalgia. Concurrently, the profound impact of environmental factors, including stress, lifestyle choices, and socioeconomic status, is well-recognized for its significant contribution to the syndrome's severity and clinical manifestations. This dual influence underscores the complexity of fibromyalgia, challenging the binary perspective of it being solely a genetic or environmental disease.

Central to our discussion has been the concept of nociplastic pain, which bridges the divide between genetic and environmental paradigms, offering a pivotal lens through which the pain experience in fibromyalgia can be understood. The recognition of nociplastic pain emphasizes the necessity of advancing our understanding beyond traditional categories, highlighting the nuanced mechanisms that contribute to the condition's pain matrix.

The call for a multidisciplinary approach in tackling fibromyalgia has never been more pressing. The integration of insights from genetics, epigenetics, the microbiome, and advancements in AI and ML, alongside the perspectives of clinical and psychological sciences, presents a promising pathway toward deciphering fibromyalgia's complexities. This collaborative effort is essential not only for the development of diagnostic tools and therapeutic interventions but also for fostering a holistic understanding of patient experiences.

Continued research and innovation are paramount in unraveling the mysteries of fibromyalgia. The dynamic interplay of genetic and environmental factors invites a broader inquiry into how these dimensions converge to influence individual susceptibility and disease trajectory. The exploration of novel methodologies, including the use of the RDoC framework and the potential of precision medicine, offers exciting avenues for advancing our knowledge and treatment approaches.

As we reflect on the question posed at the outset of this review—“Fibromyalgia: Are You a Genetic or an Environmental Disease?”—it becomes evident that the answer lies not within a binary framework but within the intricate tapestry of biological, psychological, and environmental threads that weave the fabric of this condition. Fibromyalgia embodies the complexity of human health, where the lines between genetics and environment blur, highlighting the imperative for an integrative research approach that transcends disciplinary boundaries. By fostering interdisciplinary partnerships and leveraging innovative technologies, we stand on the cusp of transformative breakthroughs in understanding, diagnosing, and treating fibromyalgia.

## Disclosures

The author has no conflicts of interest to declare.
